# Supplementary material for: Biobased Cryogels from Enzymatically Oxidized Starch: Functionalized Materials as Carriers of Active Molecules
Source: Molecules. 2020 May 31;25(11):2557. doi: 10.3390/molecules25112557 (PMC7321214; doi:10.3390/molecules25112557)
Supplement: Supplementary file 1 [file molecules-25-02557-s001.pdf]

Biobased cryogels from enzymatically oxidized starch: functionalized materials as carriers of active molecules

Antonella Caterina Boccia[1*], Guido Scavia[1], Ilaria Schizzi[2], Lucia Conzatti[2]

[1] Institute for Chemical Sciences and Technologies-SCITEC "G. Natta", CNR, Via Corti, 12, 20133 Milano, Italy; antonella.boccia@scitec.cnr.it; guido.scavia@scitec.cnr.it;

[2] Institute for Chemical Sciences and Technologies-SCITEC "G. Natta", CNR, Via De Marini, 6, 16149 Genova, Italy; lucia.conzatti@cnr.it; ilaria.schizzi@scitec.cnr.it.

* Correspondence: antonella.boccia@scitec.cnr.it; Tel.: +39 02 236 99 212

Table S1. Peculiar chemical shifts for ^{1}H species of modified PS

^{1}H	Chemical shift[1]
H-1	5.11
H-2	4.76
H-3	5.16
H-4,5	3.90 – 4.10
H-6	4.56 – 4.60
H_{ald}	9.23-9.28

[1] Chemical shifts are expressed in ppm

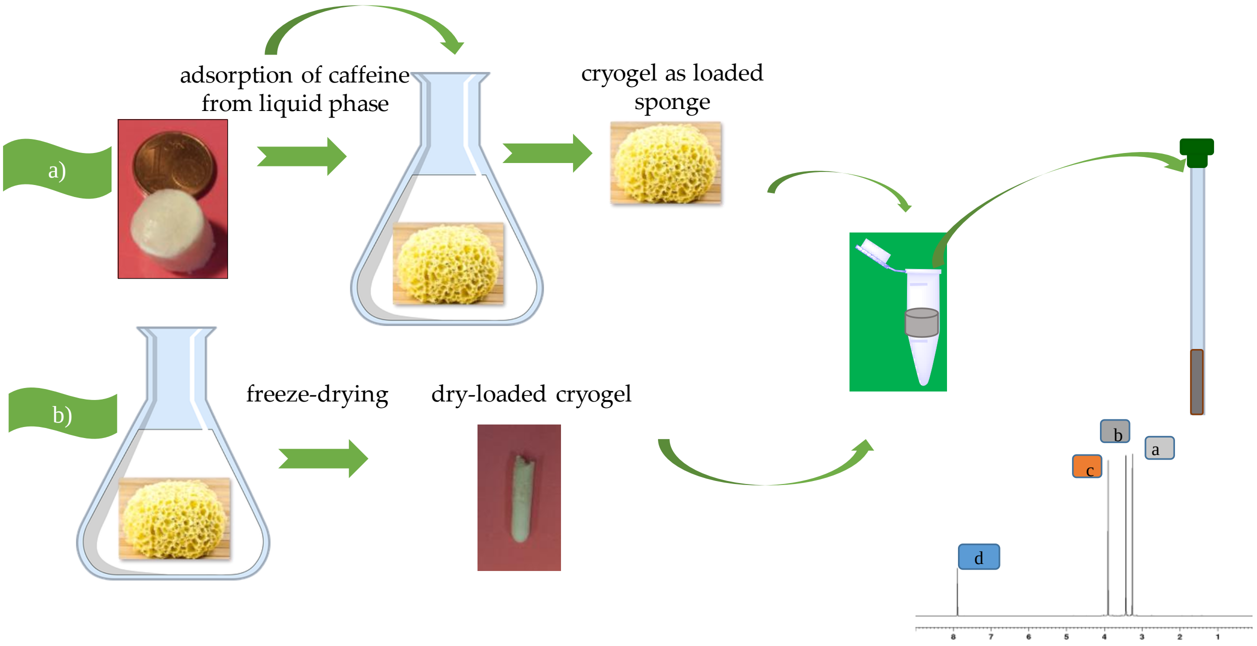

Figure S1. Experimental scheme illustrating the procedure for sorption/desorption of caffeine.

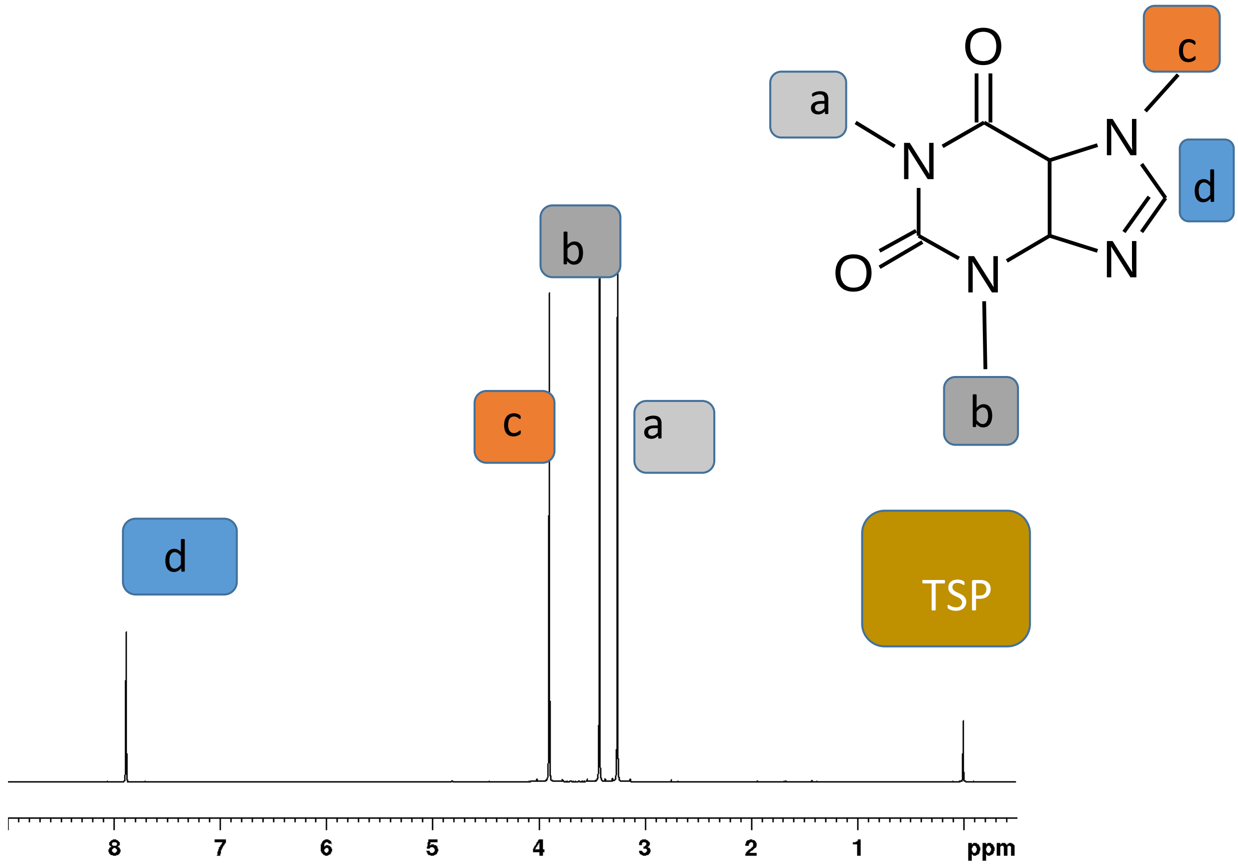

Figure S2. ^{1}H NMR spectrum of caffeine, recorded at 298 K, in D_2O.

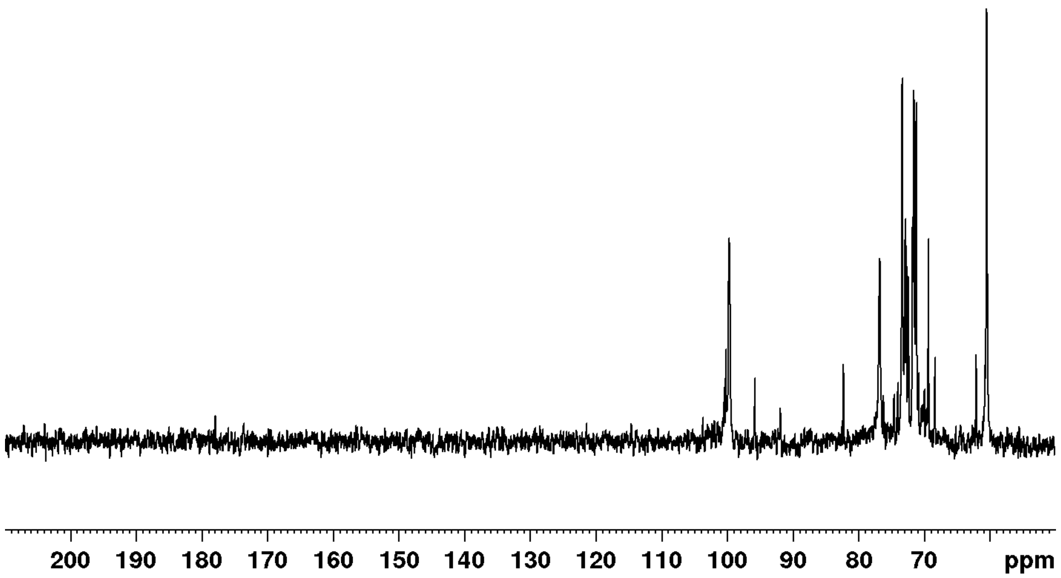

Figure S3. ^{13}C NMR spectrum of partially oxidized pea starch, (sample C), recorded at 298 K, in D_2O.

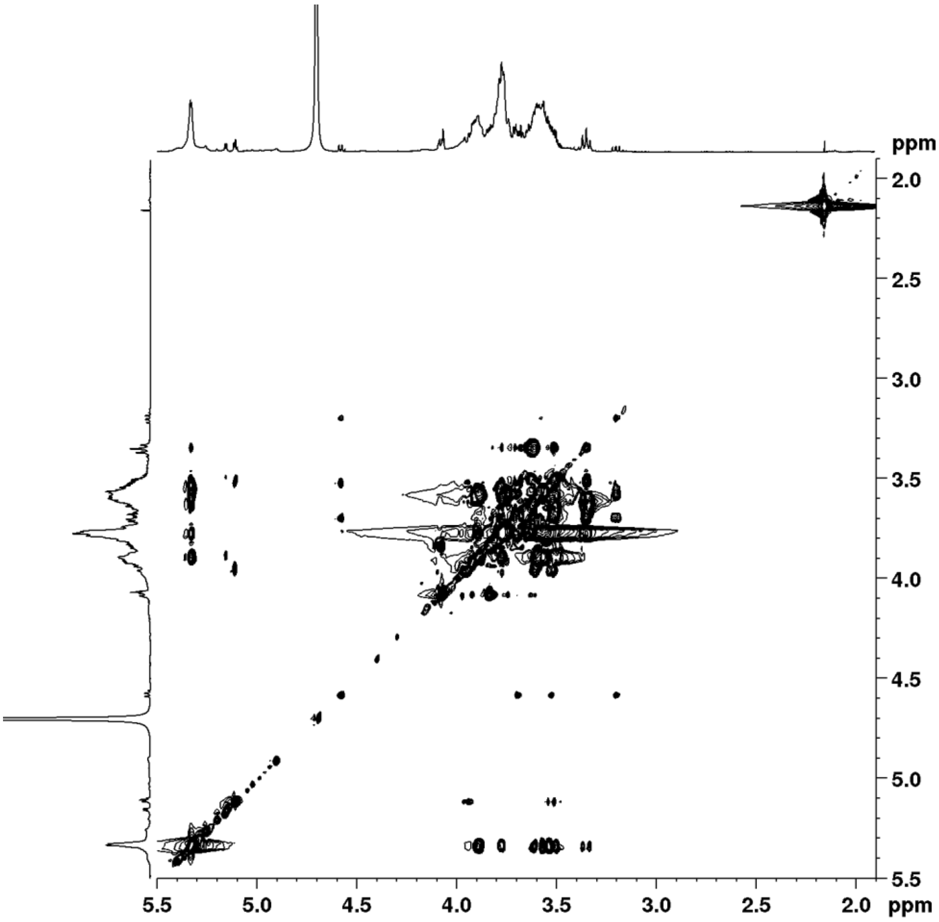

Figure S4. ^1H-^1H TOCSY spectrum of partially oxidized pea starch, (from sample C), recorded at 298 K, in D_2O.
